# Prognostic Capabilities of Diagnosing Non-Alcoholic Hepatic Steatosis at Early Stages of Its Development

**DOI:** 10.17691/stm2022.14.4.03

**Published:** 2022-07-29

**Authors:** G.I. Akhmadullina, I.А. Kurnikova

**Affiliations:** Associate Professor, Department of Intermediate Level Therapy with the Course of Endocrinology and Hematology; Izhevsk State Medical Academy, 281 Kommunarov St., Izhevsk, 426034, Russia;; Professor, Department of Hospital Therapy with the Courses of Endocrinology, Hematology, and Clinical Laboratory Diagnosis; Peoples’ Friendship University of Russia, 6 Miklukho-Maklaya St., Moscow, 117198, Russia ; Head of the Endocrinology Course; Peoples’ Friendship University of Russia, 6 Miklukho-Maklaya St., Moscow, 117198, Russia

**Keywords:** hepatic steatosis, dynamic hepatobiliscintigraphy, prognostic markers

## Abstract

**The aim of the study** is to substantiate the efficacy of new prognostic criteria in diagnosing non-alcoholic hepatic steatosis at early stages of its development.

**Materials and Methods:**

Patients with excessive body mass (n=26) and obesity (n=28) having different concomitant pathologies of the digestive organs have undergone a comprehensive examination including assessment of biochemical blood indices, ultrasound examination of the abdominal organs, dynamic hepatobiliscintigraphy, assessment of the comorbidity level as a whole (calculation of CIRS) and by the available pathology of the digestive organs.

**Results:**

The ultrasound signs of hepatic steatosis have been found in all patients with obesity stage II and III, in 69.2% of the examined patients (95% CI: 42.37–87.32) with obesity stage I, in 30.8% (95% CI: 16.50–49.99) with excessive body mass, and in 13.9% of patients (95% CI: 6.08–28.66) with normal body mass. According to the ROC analysis, the predictors of hepatic steatosis development in patients with excessive body mass and obesity are BMI (>31, р<0.0001) and the number of digestive organ illnesses (more than 4 diseases, p<0.0001). On the basis of the data obtained, a logistic model has been developed in the form of the regression equation permitting us to predict the groups of patients with a low or high degree of risk of hepatic steatosis. The analysis of dynamic hepatobiliscintigraphy has revealed deceleration of the absorbing function of hepatocytes in patients with normal ultrasound images of the liver even in case of excessive body mass. The obtained results made it possible to develop a method of diagnosing fatty hepatosis with subsequent calculation of the functional hepatocyte activity index (FHAI) which helps not only establish functional disorders but identify the group of patients having the risk of developing these disorders. Normal ultrasound imaging of the liver and absence of the biochemical changes in the blood are not indicators of preservation of its functional activity, since the risk of functional disorders was found in 32.3% of cases (95% CI: 18.57–49.86) and reversible disorders in 19.3% (95% CI: 9.19–36.28) when FHAI was calculated for patients with normal body mass. With the increase of body mass, irreversible functional disorders of hepatocytes are observed in 80–100% of patients.

Thus, the results obtained in the course of the investigation have confirmed the possibility of using new prognostic markers of hepatic steatosis for early diagnosis of non-alcoholic fatty liver disease.

## Introduction

Early diagnosis of non-alcoholic fatty liver disease (NAFLD) has been still difficult since clinical manifestations and changes in biochemical blood indices are usually absent at the stage of steatosis, and alterations of the echostructure and echogenicity typical for fatty liver infiltration during ultrasound examination can be detected only when over 30% of fat is accumulated in the liver [1, 2]. However, ultrasound examination of the liver in patients with obesity is less informative as the sensitivity and specificity of the method decrease with the increase of the body mass index (BMI) and steatosis degree [3]. Today, magnetic resonance tomography, which allows the establishment of hepatic steatosis with the liver containing more than 5.56% of fat, and puncture biopsy are considered modern diagnostic techniques [4]. The level of lipid accumulation in the form of triglycerides at the volume of more than 5% of the hepatocyte mass is believed to be indicative of steatosis [3]. Non-invasive methods (FibroScan, magnetic resonance elastography) allow for the detection of liver fibrosis but are not informative at BMI≥28 [2-4]. In this connection and with the increasing number of people with excessive body mass, the search for new approaches to early NAFLD diagnosis and improvement of methods for its prevention became a long pending issue.

**The aim of the study** is to substantiate the efficacy of new prognostic criteria in diagnosing non-alcoholic hepatic steatosis at early stages of its development.

## Materials and Methods

Twenty-six patients with excessive body mass and 28 patients with various degrees of obesity (13 patients with obesity stage I, 11 with stage II, and 4 with stage III) having concomitant pathologies of digestive organs (chronic pancreatitis, cholelithiasis, chronic gastritis, chronic duodenitis, gastroesophageal reflux disease, chronic colitis) underwent one-stage single-center examination. The comparison group was composed of patients with normal body mass (n=36) suffering also from digestive diseases (Table 1).

**Table 1:** Characteristic of patients with various body masses, Me [Q1; Q3]

Parameters	Normal (n=body 36) mass	Excessive (n=body 26) mass	Obesity (n=28)	р1	р2	р3
Age (years)	40.0 [36.97; 45.0]	49.50 [43.55; 52.45]	43.50 [40.0; 46.64]	0.031	0.329	0.085
Sex (male/female)	34/2	26/0	28/0	—	—	—
Body mass index	22.85 [22.26; 23.40]	27.20 [26.31; 28.09]	35.40 [33.40; 36.0]	<0.0001	<0.0001	<0.0001
Comorbidity index	4.0 [4.0; 5.0]	4.0 [4.0; 5.0]	5.0 [5.0; 6.0]	0.889	0.032	0.0154
CIRS (points)	10.0 [8.0; 12.0]	12.0 [10.0; 13.0]	11.0 [8.0; 12.64]	0.0972	0.5193	0.4445
Fasting plasma glucose (mmol/l)	4.85 [4.50; 5.10]	4.80 [4.15; 5.80]	5.50 [4.80; 6.19]	0.913	0.056	0.170
Total cholesterol (mmol/l)	4.30 [3.86; 4.74]	4.60 [4.04; 4.88]	5.10 [4.49; 5.52]	0.525	0.039	0.119
ALT (U/l)	21.0 [18.0; 24.76]	21.0 [18.0; 28.71]	29.50 [20.0; 34.45]	0.787	0.117	0.262
АСТ (U/l)	24.50 [21.0; 29.0]	23.0 [17.0; 26.65]	25.0 [19.29; 33.12]	0.253	0.828	0.176

Note: р_1_ is statistical significance of differences between the groups having normal and excessive body mass; р_2_ — between the groups with normal body mass and obesity; р_3_ — between the groups with excessive body mass and obesity.

The study complies with the Declaration of Helsinki (2013) and was approved by the Bioethics Committee of Izhevsk State Medical Academy (Russia). Written informed consent was obtained from each patient.

Patients with renal failure (chronic kidney disease C4 and C5), viral hepatitis, markers of autoimmune liver diseases and accumulation diseases, and those taking hepatotoxic alcohol doses were excluded from the study. General clinical examination has been conducted to all patients according to the existing diagnostic standard. Of the biochemical parameters, fasting plasma glucose (FPG) test, total cholesterol, alanine aminotransferase (ALT), aspartate aminotransferase (AST) were determined. MyLab Seven system (Esaote, Italy) was used for ultrasound liver examination. The degrees of hepatic steatosis were determined according to the Batzkov’s classification of fatty liver dystrophy (1996). The absorbing and excretory function of the liver was tested by dynamic hepatobiliscintigraphy using radiopharmaceutical Bromeside, ^99m^Тс (with a standard choleretic breakfast: two raw egg yolks) with the help of Symbia T16 scintillation gamma-camera (Siemens, Germany). Subsequent analytical processing of the results was performed using the SUPER-SEGAMS computer system (Hungary). The comorbidity level was evaluated by CIRS (Cumulative Illness Rating Scale). Additionally, the comorbidity index was calculated for each patient taking into consideration the existing pathology of the digestive organs.

### Statistical data analysis

The obtained data were statistically processed using the Statistica 10.0 software package and MedCalc 12.5.0.0 program. The Shapiro– Wilk test was applied to check the normality of real data distribution. Values which did not follow the normal distribution were presented as the median, upper and lower quartiles, i.e. Me [Q1; Q3]; relative — as the frequency of attribute occurrence and 95% confidence interval (CI) calculated by the Wilson method. To calculate statistically significant differences between the quantitative variables of the independent groups, the method of multiple comparisons was employed. The critical level of significance according to the Bonferroni correction was р≤0.017. Statistical significance of differences between the qualitative variables of the independent groups was calculated using Pearson’s c^2^ test with a Yates’ correction. Mathematical modeling by building 3D plots was employed for determining the value of connection between the phenomena.

Prognostic significance of the parameters was established with the help of the ROC (receiver operating characteristic) analysis. The results were quantitatively interpreted by the ROC curves with the assessment of the AUC value. Binary logistic regression analysis was used to predict the risk of hepatic steatosis development. The binary variable was considered as a response where 0 designated absence of the predictable state (hepatic steatosis), 1 denoted its presence. In order to validate the linear regression model, the Wald test was applied. The Hosmer–Lemeshow test was used to determine how well the model fitted the real data. The goodness-of-fit hypothesis of our model was accepted at p>0.05.

## Results

The comparative analysis of the biochemical parameters has shown absence of statistically significant differences between patients with obesity and those with normal body mass (see Table 1).

According to the ultrasound findings, prevalence of hepatic steatosis expectedly increased with the increase of the body mass and was observed in all patients with obesity stage II and III. In case of obesity stage I (n=13), the signs of hepatic steatosis were found in 69.2% of cases (95% CI: 42.37–87.32), at excessive body mass (n=26) in 30.8% of cases (95% CI: 16.50–49.99), at normal body mass (n=36) in 13.9% of cases (95% CI: 6.08–28.66).

The obtained results processed with the most informative, highly sensitive, and specific ROC analysis have shown that BMI (p<0.0001) and the quantity of digestive diseases (p<0.0001) appeared to be predictors of hepatic steatosis in patients with excessive body mass and obesity (Table 2).

**Table 2:** Characteristic of hepatic steatosis predictors

Parameters	Cut-off threshold	Sensitivity (%)	Specificity (%)	Area under curve	p
Body mass index	>31	69.6	89.5	0.852	<0.0001
Comorbidity index	>4	82.6	73.7	0.829	<0.0001

The data obtained in the process of the study allowed us to develop a logistic model with the greatest concordance coefficient and the logistic regression equation:

P=exp(−16.557+0.345⋅X1+1.313⋅X2)1+exp(−16.557+0.345⋅X1+1.313⋅X2),

where *P* is the probability of steatosis development in patients with excessive body mass and obesity; exp is the base of natural logarithms 2.71…; 0.345 and 1.313 are regression coefficients; 16.557 is an intercept term (the constant); *Х*1, *Х*2 are the terms of the binary logistic regression (Table 3).

**Table 3:** Assessment of variables in the binary logistic regression equation

Parameters	Variable designation	Regression coefficient	Standard error	Wald statistic	р	Odds ratio, exp(B)	95% CI
Body mass index	*Х*1	0.345	0.125	7.569	0.006	1.412	1.095–1.819
Comorbidity index	*Х*2	1.313	0.572	5.271	0.021	3.718	1.169–11.827
Constant	—	–16.556	5.078	10.630	0.001	—	—

Analyzing the data from Table 3, we have established that increase of BMI only by one unit increases the probability of hepatic steatosis by 1.41-fold, while increase of the comorbidity index by one disease results in 3.72-fold increase.

**Figure 1.**
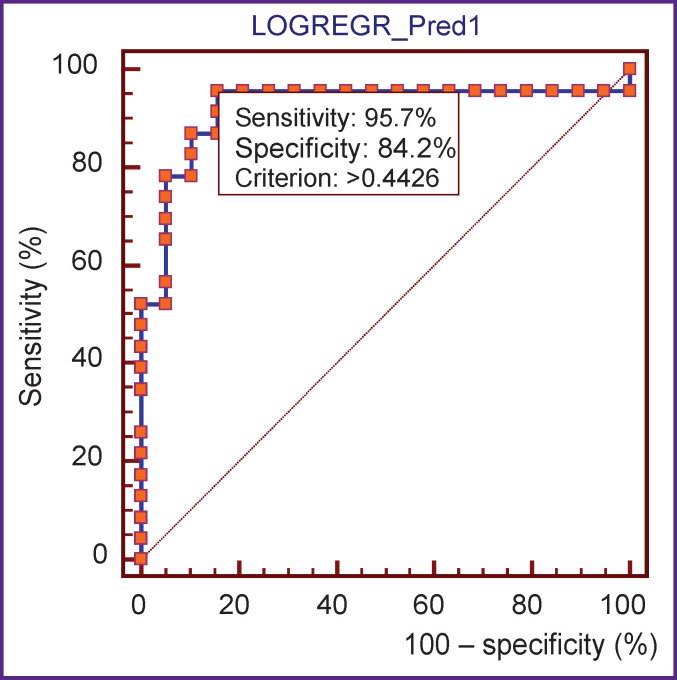
ROC curve plot for the assessment of model efficacy

The ROC analysis, aimed at determining the sensitivity and specificity, has shown that the sensitivity of the proposed model makes up 95.7%, the specificity — 84.2% (Figure 1). When building the ROC curve plot, the calculated area under the curve (AUC) was equal to 0.919 (95% CI: 0.793–0.981; p<0.0001), which corresponds to the “excellent” quality of the model according to the expert AUC model.

While building the ROC curve, a cut-off point equal to 0.4426 was obtained. It means that if *Р* is equal or exceeds 0.4426, the probability of steatosis occurrence is high, if it is less than 0.4426, the probability is low (see Figure 1). The comparison of the results predicted by the logistic regression with real data has revealed a high percentage of their fit (85.2%). Validation of the hypothesis about the adequacy of the real and predicted values with the help of the Hosmer–Lemeshow test has shown the level of significance equal to 0.385 which allowed us to make the conclusion about the goodness of fit of the proposed model.

The analysis of dynamic hepatobiliscintigraphy results has demonstrated statistically significant deceleration of time of achieving maximal accumulation of radiopharmaceutical in the liver (T_max_) and the rate of its elimination from the liver parenchyma (Т_1/2_) in patients with excessive body mass (T_max_ was 16.0 (14.0– 18.0) min, р=0.003 and T_1/2_ — 31.2 (21.7–66.0) min, р=0.013) and obesity stage I (T_max_ was 16.0 (14.5– 19.5) min, р=0.003 and T_1/2_ — 29.9 (26.8–37.2) min, р=0.011) compared to the examined patients with normal body mass (T_max_ was 12.5 (10.7–15.0) min and Т_1/2_ — 23.8 (19.4–29.4) min). Patients with obesity stage II were observed to have the tendency to the improvement of these parameters, while patients with obesity stage III had the tendency to the deceleration of the absorbing and excretory liver function.

Making the in-depth analysis of the radioisotope investigation, it has been established that patients with invariable imaging of the liver have been noted to have a tendency to slowing-down its absorbing and excretory functions already at excessive body mass (Table 4). This fact confirmed the liver damage in patients with excessive body mass and obesity prior to the appearance of the ultrasound signs characteristic of steatosis.

**Table 4:** Indices of dynamic hepatobiliscintigraphy for examined patients with normal ultrasound liver imaging depending on body mass, Me [Q1; Q3]

Parameters	Normal body mass (n=31)	Excessive body mass (n=15)	Obesity stage I (n=3)	Obesity stage II (n=1)	Obesity stage III (n=0)
T_max_ (min)	12.5 [11.1; 15.2]	16.0 [13.2; 17.9] (р=0.024)	15.0 [15.0; 16.1] (р=0.153)	10.0	—
Т_1/2_ (min)	24.1 [19.6; 32.5]	32.8 [22.2; 62.3] (р=0.043)	37.2 [31.3; 37.2] (р=0.064)	16.8	—

Note: p is statistical significance of differences in the values in comparison with the data for patients with normal body mass.

**Figure 2.**
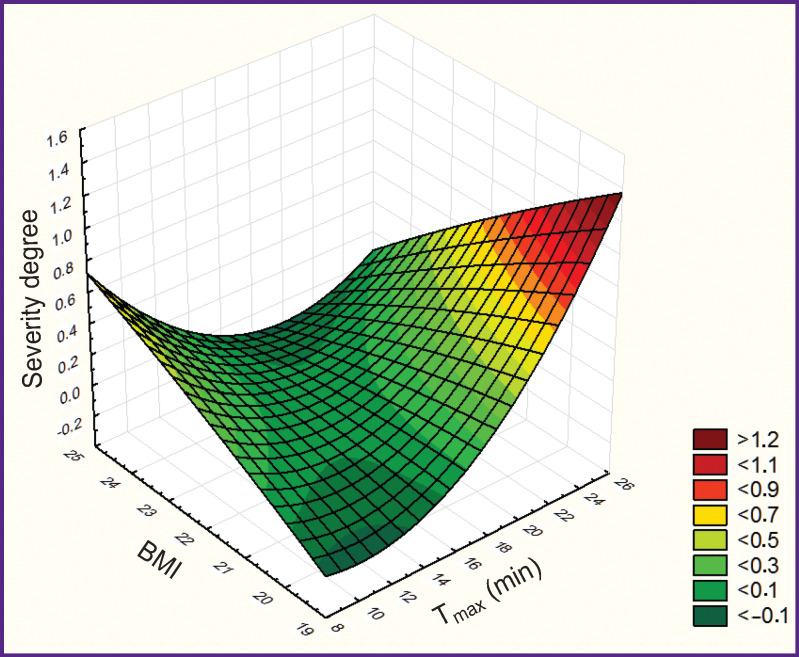
Correlation between the degree of the liver damage severity, BMI, and Tmax

Mathematical modeling with 3D plot building has revealed association of the degree of fatty liver infiltration (according to Batzkow, 1996) with BMI and the absorbing liver function. The degree of fatty infiltration grows with the increase of the patient’s body mass and deceleration of the absorbing function (Figure 2).

The results of the study allowed us to propose the formula for early identification of hepatic steatosis:

FHAI = (−1.1564+0.0653⋅BMI−0.0144⋅Tmax)⋅100,

where FHAI is functional hepatocyte activity index; BMI is body mass index; T_max_ is an indicator of the liver absorbing function (min).

The conclusion on the functional hepatocyte activity was based on the index value obtained: 0–9.9 was considered normal functional activity of hepatocytes; 10– 19.9 indicated the risk of functional disorders; 20–29.9 signified reversible disorders of the hepatocyte function (steatosis); over 30 was for irreversible (organic) disturbances of the liver function [5].

FHAI calculation for cases with normal ultrasound liver images and without biochemical blood changes has shown that patients with normal body mass (n=31) had normal functional hepatocyte activity in 35.5% of cases (95% CI: 21.12–53.05), the risk of disorder development in 32.3% (95% CI: 18.57–49.86), and reversible functional disorders in 19.3% (95% CI: 9.19–36.28). In 12.9% of the examined patients (95% CI: 5.13–28.85), the values of FHAI<0 have been obtained due to the incorrect procedure of dynamic hepatobiliscintigraphy (intake of drugs or food before the examination). The body mass increase resulted in irreversible functional disorders of the liver in 80– 100% of patients. Among the patients with excessive body mass, 20% (95% CI: 7.05–45.19) had reversible functional disorders; in 80% (95% CI: 54.81–92.95), the disorders were irreversible according to the FHAI level. All patients with obesity were found to have irreversible functional disorders as seen by FHAI calculations. Altered ultrasound imaging of the liver and biochemical blood indices were observed in all patients with obesity stage III.

## Discussion

Non-alcoholic fatty liver disease at the stage of steatosis usually runs asymptomatically, is most often detected accidentally, and is of benign character, that is why many investigations are devoted to the methods of diagnosing this disease (especially non-invasive) [3, 4, 6–9]. According to the clinical recommendations for the diagnosis and treatment of NAFLD, all patients with insulin resistance and/or metabolic risk factors as well as those vulnerable to a high risk of cardiovascular diseases should be examined for NAFLD. At the same time, it is well known that up to 22% of patients with NAFDL do not even have excessive body weight [2, 3]. The results of our study have demonstrated that ultrasound signs of steatosis were revealed in 13.9% of individuals with normal body mass.

A great number of factors play a role in the NAFLD pathogenesis including chronic diseases of the gastro-intestinal tract with secretory insufficiency of digestive enzymes and impairment of digestive processes and absorption, the syndrome of excessive bacterial growth and/or intestinal dysbiosis [7, 9–11]. In our study, we have established that the number of digestive diseases may be considered to be the marker of hepatic steatosis.

The existing non-invasive tests for the assessment of hepatic steatosis such as NLFS test, HSI, and FLI make it possible to identify presently existing hepatic steatosis in contrast to the method of FHAI calculation, the values of which provide the possibility to diagnose hepatic steatosis prior to the appearance of ultrasound changes typical for fatty liver infiltration, define patients with the risk of functional disorders, and determine the reversibility of these disorders. To calculate FHAI, it is necessary to count only one parameter during dynamic hepatobiliscintigraphy, T_max_, thereafter, the examination may be terminated reducing thereby the radiation dose and examination time.

## Conclusion

The results of our work have shown that the developed logistic model is an effective tool for determining the probability of forming non-alcoholic fatty liver disease in patients with excessive body mass or obesity and comorbid pathology of the digestive system. Once a high probability of forming non-alcoholic steatosis has been revealed, dynamic hepatobiliscintigraphy should be conducted with subsequent calculation of the functional hepatocyte activity index to define more exactly the reversibility of the hepatocyte functional disorders and to work out pathogenetic methods for their prevention and management.
